# Association between the dietary omega-6 to omega-3 fatty acid ratio and age-related macular degeneration in Korean adults

**DOI:** 10.1186/s12937-025-01090-z

**Published:** 2025-03-09

**Authors:** Won Jang, Yuna Kim, Hyesook Kim

**Affiliations:** 1 Department of Food and Nutrition, Wonkwang University, 460 Iksandae-ro, Iksan, Jeonbuk-do Republic of Korea; 2 Institute for Better Living, Wonkwang University, 460 Iksandae-ro, Iksan, Jeonbuk-do Republic of Korea; 3 Department of Clinical Nutrition Science, The Graduate School of Clinical Health Sciences, Ewha Womans University, 52 Ewhayeodae-gil, Seodaemun-gu, Seoul, Republic of Korea

**Keywords:** Age-related macular degeneration, Polyunsaturated fatty acids, Omega-6, Omega-3, Korean national health and nutrition examination survey

## Abstract

**Background:**

Long-chain polyunsaturated fatty acids of the omega-6 and omega-3 families affect processes implicated in vascular and neural retinal disease pathogenesis. This study aimed to investigate the association between the dietary omega-6 to omega-3 fatty acid ratio and age-related macular degeneration (AMD).

**Methods:**

We conducted a cross-sectional analysis using a nationwide representative sample of older adults (≥ 50 years), including 1,944 men and 2,592 women, from the Korea National Health and Nutrition Examination Survey (2017–2018). Omega-6 and omega-3 fatty acid intakes were collected through a 24-hour recall method and used to calculate the omega-6 to omega-3 fatty acid ratio. Associations between the ratio and AMD were determined using odds ratios (ORs) from multivariate logistic regressions.

**Results:**

The prevalence of AMD was 19.8% and 17.7% in Korean men and women, respectively. In women, the multivariable-adjusted OR for incurring AMD was significantly higher in the 2nd (OR = 1.36; 95% CI = 1.02–1.81) and 3rd (OR = 1.36; 95% CI = 1.02–1.83) tertiles of the dietary omega-6 to omega-3 fatty acid ratio than in the 1st tertile (OR = 1, the reference OR) (*P* = 0.036 for this trend). However, this association was not observed in men.

**Conclusions:**

These results suggest that high omega-6 to omega-3 fatty acid ratios may be associated with an increased prevalence of AMD among Korean women.

**Supplementary Information:**

The online version contains supplementary material available at 10.1186/s12937-025-01090-z.

## Background

Age-related macular degeneration (AMD) is one of the most common eye diseases that cause vision impairment and blindness among people aged 50 and older [1–4]. Characterized by the progressive degeneration of the macula, the central part of the retina responsible for sharp, central vision, AMD affects approximately 8.7% of the global population [5]. According to a recent study, there will be 196 million people with macular degeneration in 2020, and it is predicted that the number will increase to 288 million in 2040 and continue to rise [5]. Despite its widespread impact, the exact pathophysiology of AMD is not fully understood. It is believed to result from complex interactions between metabolic, genetic, and environmental factors. Many studies have explored factors associated with AMD prevention and progression, including age [6], smoking [7, 8], alcohol consumption [9, 10], family history [11], chronic diseases [12–14], diet [15], and lifestyle [16, 17]. Among these, nutrition plays a particularly crucial role in the prevention and progression of AMD.

Antioxidant intake [18–21] and fish consumption [22–27] have emerged as notable nutritional factors associated with AMD risk reduction. Antioxidant nutrients, such as lutein and zeaxanthin, play a crucial role in protecting the macula from damage by scavenging harmful free radicals [28]. Studies investigating the Mediterranean diet, a diet rich in antioxidant foods with fish as a main component, have consistently shown its potential benefits for AMD prevention [29–32]. The consumption of lipids, particularly the omega-3 fatty acids, docosahexaenoic acid (DHA) [7, 24, 33–36], and eicosapentaenoic acid (EPA) [21, 37–39], has been extensively studied in relation to AMD. However, recent research has shifted focus towards assessing the overall balance of fatty acids, specifically the omega-6 to omega-3 fatty acid ratio. This ratio has been linked to various chronic diseases, including vascular disease [40], obesity [41, 42], inflammatory disease [43, 44], and diabetes [45]. Notably, studies have suggested that higher omega-6 to omega-3 fatty acid ratios may be associated with an increased risk of AMD [46, 47].

Despite these findings, no studies have specifically evaluated the association between the dietary omega-6 to omega-3 fatty acid ratio and AMD within Asian populations. In Asian populations, people traditionally consume significant amounts of seafood [47]. Consequently, the omega-6 and omega-3 fatty acid intake profiles may differ between Asian and Western populations. Thus, the present study aimed to investigate the relationship between dietary omega-6 to omega-3 fatty acid ratio and AMD in Koreans aged 50 years or older.

## Methods

### Study participants

This study was conducted based on the 7th Korea National Health and Nutrition Examination Survey (KNHANES; 2016–2018). In this survey, the data for 2016 did not investigate macular degeneration. Therefore, this study analyzed KNHANES data from the years 2017 and 2018. This study focused on individuals in South Korea aged ≥ 50 years. Among the 16,119 participants, subjects under 50 years in age (*n* = 9,048), with implausible energy intakes (< 500 kcal or > 5000 kcal) (*n* = 854), or lacking data on age-related macular degeneration (*n* = 1,681) were excluded. Finally, a total of 4,536 subjects (1,944 men and 2,592 women) were included in the analysis. The KNHANES received approval from the Institutional Review Board (IRB) of the Korea Disease Control and Prevention Agency (IRB no. 2018-01-03-P-A). All individuals provided informed consent before enrollment.

### Assessment of AMD

Ophthalmic retina and glaucoma tests were conducted for adults over 40 years old, and the presence or absence of AMD was assessed. A comprehensive ophthalmologic evaluation was performed by ophthalmologists dispatched by the Korean Ophthalmological Society (KOS) using a mobile unit equipped with specialized ophthalmic devices. Digital fundus photographs were captured utilizing a TRC-NW6S digital fundus camera (Topcon, Tokyo, Japan), producing a 45° retinal fundus image centered on the maculae and foveae of each participant’s eyes. Initial grading of the fundus photographs was conducted by trained ophthalmologists following the guidelines provided by the International Age-Related Maculopathy Epidemiological Study Group. Subsequently, detailed grading was undertaken by nine retinal specialists authorized by the KOS. Final grades were determined based on this detailed assessment, with any discrepancies between preliminary and detailed grades resolved by a single retinal specialist. The diagnosis of AMD, defined as the presence of soft indistinct or reticular drusen and hard or soft distinct drusen with pigmentary abnormalities, was indicated with a “yes” or “no” response in the survey questionnaire.

### Dietary assessment

Daily food and nutrient intake were evaluated utilizing data from the KNHANES 24-hour recall. Participants provided detailed reports of their food and beverage consumption from the previous day through face-to-face interviews. Food items in the dataset were categorized into 18 food groups, including cereals and cereal products, potatoes and starches, sugars and sugary products, beans and bean products, nuts and seeds, vegetables, mushrooms, fruits, meat and meat products, eggs and egg products, fish and shellfish, seaweeds, milk and dairy products, oils and fats, beverages and alcohols, seasonings, processed foods, and others. The assessment aimed to estimate energy intake and the intake levels of 16 nutrients, namely carbohydrates, protein, fat, fiber, sugar, calcium, phosphorus, iron, sodium, potassium, vitamin A, carotene, thiamin, riboflavin, niacin, and vitamin C. Dietary fatty acids can be separated into three categories: saturated fatty acids (SFAs), monounsaturated fatty acids (MUFAs), and polyunsaturated fatty acids (PUFAs). Additionally, PUFAs have 2 main components with relevant biological functions: omega-3 fatty acids, and omega-6 fatty acids. Using this data, we calculated omega-6 to omega-3 fatty acid ratios and then divided the ratio data into tertiles for further analysis: for men, Tertile 1 was 0.3–4.4, Tertile 2 was 4.5–7.3, and Tertile 3 was > 7.4 and for women, Tertile 1 was 0.3–4.2, Tertile 2 was 4.3–7.0, and Tertile 3 was > 7.1. Only dietary intake of these fatty acids was considered, with supplements not being taken into account.

### Assessment of other variables

This study obtained general information about the demographic and socioeconomic characteristics of participants, including age, body mass index (BMI), income, education level, marital status, disease diagnoses, menopausal status (women only), stress status, alcohol consumption, smoking status, physical activity status, supplement usage, energy intake, beta-carotene intake, and vitamin C intake, which was collected by trained interviewers. Monthly family income was divided into four groups (“low,” “middle-low,” “middle-high,” and “high”). Education level was set into four groups (“elementary school graduation,” “middle school graduation,” “high school graduation,” and “university graduation and above”). Marital status was divided into two categories, “single” and “married” (including widowed and divorced). Disease diagnoses were classified as “yes” or “no,” and for women, menopausal status was recorded. Stress level was categorized as “high” or “low” based on the respondent’s self-evaluation. Alcohol consumption was categorized as “yes” if the participant drank at least once a month, and for smoking status, respondents were designated as smokers if they smoked more than 5 packs of cigarettes in their lifetime. The physical activity status of responders was categorized as “yes” or “no” based on whether they participated in medium-intensity physical activity for more than 2.5 h a week, high-intensity physical activity for more than 1.25 h a week, or both medium- and high-intensity activities adding up to equivalent times. Lastly, dietary supplement usage was classified as “yes” if supplements were consumed in the past year or “no” otherwise.

### Statistical analysis

Following the guidelines from the Korea Centers for Disease Control and Prevention regarding the utilization of national health and nutrition survey data, complex sample data analysis was conducted considering weights, stratified variables, and collective variables to represent the entire country using the SURVEYMEANS and SURVEYFREQ procedures in SAS (version 9.4; SAS Institute, Cary, NC, USA). The SURVEYREG procedure was used to analyze the means, general characteristic distributions, and total differences between men and women using independent t-tests for continuing variables and chi-square tests for categorical variables.

The omega-6 to omega-3 fatty acid ratios of the subjects’ daily dietary intake were divided into tertiles. A logistic regression analysis was performed to obtain the odds ratio (OR) of suffering AMD according to the omega-6 to omega-3 fatty acid ratio. Covariates were chosen based on their statistical significance in univariate analyses or because they are known risk factors associated with the relationship between fatty acid intake and AMD in the existing literature. Thus, the final model was adjusted for age, BMI, monthly family income, education level, marital status, disease diagnoses, menopausal status (women only), stress status, alcohol consumption, smoking status, physical activity status, supplement usage, energy intake, beta-carotene intake, and vitamin C intake. All statistical analyses were performed using SAS (version 9.4), and all tests were two-sided with an acceptable error rate of < 0.05.

## Results

### Characteristics of the subjects in relation to the presence of AMD

Participant characteristics by tertile of the dietary omega-6 to omega-3 fatty acid ratio are displayed in Table 1. In men, the dietary omega-6 to omega-3 fatty acid ratio tended to be higher in participants who were younger (*P* < 0.001), had a higher BMI (*P* = 0.025), had a low monthly family income (*P* = 0.012), and/or suffered from chronic diseases (*P* < 0.001). Among women, participants who consumed alcohol at least once a month (*P* = 0.020), had higher education levels (*P* < 0.001), had high monthly family incomes (*P* < 0.001), smoked (*P* = 0.004), were married (*P* = 0.002), suffered from chronic diseases (*P* < 0.001), and/or had experienced menopause (*P* < 0.001) tended to have higher omega-6 to omega-3 fatty acid ratios.

**Table 1:** Participants’ general characteristics according to tertile of dietary omega-6 to omega-3 fatty acid ratio

Variables	Men				Women			
	Tertile 1^b^ (*n* = 648)	Tertile 2 (*n* = 648)	Tertile 3 (*n* = 648)	*P*-value^d^	Tertile 1 (*n* = 864)	Tertile 2 (*n* = 864)	Tertile 3 (*n* = 864	*P*-value
Age, years	63.4 ± 0.5^c^	62.1 ± 0.4	61.3 ± 0.4	< 0.001	63.7 ± 0.4^3)^	62.5 ± 0.4	63.0 ± 0.4	< 0.001
BMI^1^, kg/m^2^	24.0 ± 0.1	24.0 ± 0.1	24.5 ± 0.1	0.025	23.9 ± 0.1	23.9 ± 0.1	24.1 ± 0.1	0.533
Alcohol drinking, *n* (%)				0.209				0.020
< 1time/month	232(33.8)	203(29.7)	236(33.2)		634(71.2)	619(69.2)	628(71.2)	
≥ 1time/month	414(66.2)	444(70.3)	410(66.8)		862(28.8)	240(30.8)	230(28.8)	
Smoking, *n* (%)				0.402				0.004
Yes	172(28.7)	172(28.4)	192(31.2)		22(2.0)	22(2.5)	28(3.3)	
No	474(71.3)	474(71.6)	645(68.8)		841(98.0)	835(97.5)	457(96.7)	
Education level, *n* (%)				0.080				< 0.001
Elementary school or less	162(22.0)	144(19.0)	152(18.7)		384(40.7)	352(36.9)	341(34.4)	
Middle school	104(15.3)	101(15.1)	100(15.8)		134(17.1)	136(15.2)	124(14.8)	
High school	191(32.0)	182(30.4)	212(34.9)		201(26.9)	231(31.4)	226(31.7)	
College or above	162(30.7)	189(35.4)	159(30.6)		114(15.3)	118(16.6)	146(19.1)	
Income, *n* (%)				0.012				< 0.001
Low	150(19.2)	148(19.5)	161(20.6)		255(25.6)	264(25.7)	245(24.4)	
Middle-low	191(27.4)	163(23.2)	164(23.5)		234(26.2)	217(26.0)	217(25.2)	
Middle-high	152(24.6)	145(22.9)	157(25.2)		184(23.0)	194(24.5)	195(24.4)	
High	163(28.9)	190(34.4)	163(30.6)		189(25.2)	186(23.7)	206(26.0)	
Marital status, *n* (%)				0.220				0.002
Single	10(1.5)	19(2.9)	25(3.7)		12(1.2)	10(1.1)	12(1.3)	
Marital	638(98.5)	629(97.1)	623(96.3)		852(98.8)	854(98.9)	852(98.7)	
Diagnosis of disease, *n* (%)				< 0.001				< 0.001
Yes	415(58.0)	406(59.5)	409(59.5)		637(70.9)	591(64.9)	618(69.3)	
No	232(42.0)	242(40.5)	239(40.5)		227(29.1)	273(35.1)	246(30.7)	
Supplements, *n* (%)				0.567				0.312
Yes	331(51.6)	342(53.1)	311(50.1)		549(63.6)	551(62.2)	539(64.0)	
No	317(48.4)	306(46.9)	337(49.9)		315(36.4)	313(37.8)	325(36.0)	
Menopausal status, *n* (%)								< 0.001
Yes	-	-	-		689(78.2)	684(76.3)	680(77.3)	
No	-	-	-		175(21.8)	180(23.7)	184(22.7)	
Practice of aerobic exercise, *n* (%)				0.1939				0.109
Yes	220(37.4)	222(37.4)	230(37.3)		277(36.5)	288(37.6)	281(34.9)	
No	400(62.6)	393(62.6)	390(62.8)		555(63.5)	547(62.4)	55(65.1)	
Stress, *n* (%)				0.0877				0.378
High	114(19.9)	109(19.0)	121(19.9)		198(25.4)	193(21.8)	206(22.9)	
Low	532(80.1)	537(81.0)	524(80.1)		665(74.6)	665(78.2)	649(77.1)	

^a^ BMI, body mass index. ^b^ The tertile of alternative n-6 to n-3 fatty acid ratio intake.^c^ Data are presented as Mean ± SE or Number (percentage). ^d^*P*-value is for the test of difference in means of the variables by the tertile of n-6 to n-3 fatty acid ratio intake using one-way ANOVA (Analysis of Variance) for continuous variables and chi-square test for categorical variables.

### Daily nutrient intake in relation to the presence of AMD

The daily food intake of Korean older adults by tertile of the dietary omega-6 to omega-3 fatty acid ratio is presented in Table 2. In men, the intake of beans and bean products, vegetables, fish and shellfish, and seasonings was lower in the third tertile, while the intake of meat and meat products, eggs and egg products, and processed foods was significantly higher (*P* < 0.05). In women, the intake of beans and bean products, meat and meat products, eggs and egg products, and oils and fats were significantly higher in the third tertile, while the intake of nuts and seeds, vegetables, fruits, fish and shellfish, and seaweeds was lower (*P* < 0.05).

**Table 2:** Participants’ daily food intake according to tertile of dietary omega-6 to omega-3 fatty acid ratio

Variables^1^	Men				Women			
	Tertile 1^a^ (*n* = 648)	Tertile 2 (*n* = 648)	Tertile 3 (*n* = 648)	*P*-value ^c^	Tertile 1 (*n* = 864)	Tertile 2 (*n* = 864)	Tertile 3 (*n* = 864	*P*-value
Cereal and cereal products (g)	300.6 ± 6.9^b^	313.9 ± 8.0	3189.0 ± 7.8	0.180	244.0 ± 6.0	239.5 ± 4.5	254.4 ± 5.6	0.092
Potatoes and starchy products (g)	29.6 ± 3.0	37.1 ± 54.7	29.4 ± 3.1	0.251	41.4 ± 4.3	42.8 ± 4.6	36.7 ± 3.4	0.452
Sugar and sugary products (g)	5.6 ± 0.5	7.0 ± 1.4	7.0 ± 0.6	0.144	5.61 ± 0.5	4.7 ± 0.3	5.7 ± 0.4	0.142
Beans and bean products (g)	47.2 ± 5.8	66.9 ± 0.5	37.2 ± 3.1	< 0.001	31.6 ± 2.6	45.9 ± 3.3	34.8 ± 2.9	0.003
Nuts and seeds (g)	6.4 ± 0.6	6.6 ± 0.5	8.8 ± 1.1	0.157	12.1 ± 1.5	6.9 ± 0.9	9.8 ± 1.0	0.005
Vegetables (g)	397.0 ± 10.4	389.4 ± 0.3	357.3 ± 12.2	0.021	326.2 ± 9.3	309.6 ± 9.1	257.7 ± 7.3	< 0.001
Mushroom (g)	6.0 ± 0.9	5.8 ± 1.0	5.2 ± 0.9	0.826	5.6 ± 0.8	6.3 ± 1.1	4.5 ± 0.5	0.315
Fruits (g)	199.6 ± 15.1	213.2 ± 8.5	183.7 ± 13.1	0.292	278 ± 14.3	305.4 ± 12.0	221.2 ± 11.3	0.004
Meat and meat products (g)	61.8 ± 4.6	86.4 ± 8.1	138.0 ± 6.8	< 0.001	40.5 ± 3.5	59.33.8	82.2 ± 4.8	< 0.001
Eggs and egg products (g)	20.7 ± 1.7	26.2 ± 0.1	29.1 ± 2.1	0.007	17.5 ± 1.3	22.2 ± 1.3	26.9 ± 1.6	< 0.001
Fish and shellfish (g)	181.3 ± 9.9	138.9 ± 8.6	78.0 ± 6.7	< 0.001	124.5 ± 5.3	90.2 ± 5.3	60.1 ± 4.7	< 0.001
Seaweed (g)	43.5 ± 6.9	36.0 ± 31.6	31.2 ± 4.6	0.338	39.9 ± 4.2	29.6 ± 3.1	22.4 ± 2.7	0.002
Milk and dairy products (g)	61.7 ± 5.5	36.0 ± 10.5	59.8 ± 5.6	0.138	83.2 ± 5.0	81.7 ± 5.3	83.4 ± 4.9	0.966
Oil and fat (g)	5.5 ± 0.3	5.6 ± 0.3	5.8 ± 0.4	0.841	4.5 ± 0.2	3.7 ± 0.2	4.8 ± 0.3	0.002
Beverages and alcohols (g)	272.1 ± 24.1	278.2 ± 19.1	288.6 ± 22.5	0.874	131.6 ± 10.0	140.2 ± 10.0	135.1 ± 10.3	0.821
Seasoning (g)	35.5 ± 1.6	40.6 ± 1.8	35.4 ± 1.6	0.041	25.8 ± 1.0	26.4 ± 1.1	24.9 ± 1.0	0.585
Processed food (g)	6.4 ± 1.6	13.5 ± 2.8	11.8 ± 2.1	0.034	6.6 ± 1.6	5.2 ± 1.0	9.1 ± 1.6	0.126
Other (g)	0.7 ± 0.3	0.7 ± 0.3	2.0 ± 0.9	0.359	0.3 ± 0.1	0.24 ± 0.1	1.1 ± 0.7	0.121

^a^The tertile of alternative n-6 to n-3 fatty acid ratio intake. T1: 1st tertile, T2: 2nd tertile, T3: 3rd tertile. ^b^Data is presented as Mean ± SE. ^c^*P*-value is for the test of difference in means of the variables by tertiles of n-6 to n-3 fatty acid ratio using one-way ANOVA (Analysis of Variance) for continuous variables

The daily nutrient intake of middle-aged Koreans by tertile of the omega-6 to omega-3 fatty acid ratio is summarized in Table 3. All fat nutrients showed significant differences. Of these nutrients, all except omega-3 fatty acids were higher when the ratio was lower (*P* < 0.05).

**Table 3:** Participants’ daily nutrient intake according to tertile of dietary omega-6 to omega-3 fatty acid ratio

Variables^a^	Men				Women			
	Tertile 1^b^ (*n* = 648)	Tertile 2 (*n* = 648)	Tertile 3 (*n* = 648)	*P*-value ^d^	Tertile 1 (*n* = 864)	Tertile 2 (*n* = 864)	Tertile 3 (*n* = 864	*P*-value
Energy (kcal)	2157.8 ± 41.1^c^	2131.1 ± 39.8	2185.2 ± 39.2	0.637	1579.9 ± 22.7	1591.3 ± 23.7	1618.3 ± 23.1	0.399
Carbohydrate (g)	162.9 ± 1.9	157.7 ± 1.6	158.7 ± 1.6	0.087	172.8 ± 1.1	170.1 ± 1.2	172.7 ± 01.1	0.191
Protein (g)	33.7 ± 0.4	35.1 ± 0.4	35.1 ± 0.4	0.018	34.5 ± 0.4	34.7 ± 0.4	33.9 ± 0.4	0.302
Fat (g)	16.2 ± 0.3	17.8 ± 0.4	19.8 ± 0.4	0.009	17.0 ± 0.4	18.0 ± 0.3	20.0 ± 0.4	0.072
SFA (g)	4.8 ± 0.1	5.5 ± 0.1	6.6 ± 0.2	< 0.001	5.1 ± 0.1	5.7 ± 0.1	6.4 ± 0.2	< 0.001
MUFA (g)	4.7 ± 0.1	5.4 ± 0.1	6.5 ± 0.2	< 0.001	5.0 ± 0.1	5.5 ± 0.1	6.5 ± 0.2	< 0.001
PUFA (g)	5.2 ± 0.1	5.3 ± 0.1	4.7 ± 0.1	< 0.001	5.4 ± 0.1	5.2 ± 0.1	5.3 ± 0.1	< 0.001
Omega-3 PUFA (g)	1.6 ± 0.1	0.8 ± 0.0	0.5 ± 0.0	< 0.001	1.8 ± 0.1	0.8 ± 0.0	0.5 ± 0.0	< 0.001
Eicosapentaenoic Acid, EPA (mg)	161.3 ± 9.2	53.1 ± 2.6	14.5 ± 1.1	< 0.001	138.9 ± 6.3	44.1 ± 2.0	4.8 ± 0.1	< 0.001
Docosahexaenoic Acid, DHA (mg)	317.5 ± 22.0	96.0 ± 4.6	32.7 ± 2.0	< 0.001	268.0 ± 14.1	78.7 ± 3.9	7.1 ± 0.2	< 0.001
EPA + DHA (mg)	478.8 ± 30.8	149.0 ± 6.8	47.2 ± 2.9	< 0.001	407.0 ± 20.0	122.8 ± 5.6	16.1 ± 0.8	< 0.001
Omega-6 PUFA (g)	3.6 ± 0.1	4.5 ± 0.1	4.3 ± 0.1	< 0.001	3.6 ± 0.1	4.4 ± 0.1	7.2 ± 0.2	< 0.001
Linoleic Acid (mg)	3518.3 ± 75.4	4443.6 ± 95.0	4188.7 ± 105.9	< 0.001	3526.3 ± 75.9	4344.0 ± 105.5	4747.0 ± 125.5	< 0.001
Fiber (g)	14.1 ± 0.3	13.9 ± 0.3	14.2 ± 0.3	0.700	16.1 ± 0.4	16.3 ± 0.3	16.8 ± 0.4	0.340
Sugar (g)	27.2 ± 0.8	26.8 ± 0.8	28.1 ± 0.7	0.365	34.2 ± 0.9	35.2 ± 0.7	35.1 ± 0.8	0.608
Calcium (mg)	261.9 ± 5.2	274.3 ± 5.7	272.4 ± 5.9	0.177	296.0 ± 7.0	304.3 ± 6.2	299.3 ± 6.6	0.641
Phosphorus (mg)	535.0 ± 6.5	548.3 ± 6.4	552.3 ± 5.9	0.122	569.5 ± 6.8	574.8 ± 6.0	563.2 ± 5.9	0.332
Iron (mg)	6.3 ± 0.1	6.6 ± 0.1	6.7 ± 0.1	0.102	6.8 ± 0.1	6.9 ± 0.1	6.8 ± 0.1	0.789
Sodium (mg)	1793.2 ± 36.5	1881.3 ± 34.7	1762.5 ± 33.9	0.041	1725.6 ± 35.3	1709.5 ± 33.2	1726.0 ± 43.2	0.905
Potassium (mg)	1465.4 ± 25.9	1490.2 ± 21.4	1532.9 ± 22.7	0.122	1659.9 ± 26.0	1685.6 ± 21.1	1629.2 ± 22.1	0.702
Vitamin A(µgRE)	298.3 ± 11.3	306.4 ± 17.5	313.0 ± 11.1	0.618	321.1 ± 12.8	329.9 ± 14.3	358.5 ± 12.0	0.856
Carotene (µg)	1497.9 ± 63.0	1432.6 ± 52.4	1535.1 ± 65.6	0.433	1716.6 ± 67.6	1725.1 ± 60.5	1767.7 ± 66.4	0.831
Thiamin (mg)	0.7 ± 0.0	0.7 ± 0.0	0.7 ± 0.0	0.856	0.7 ± 0.0	0.7 ± 0.0	0.7 ± 0.0	0.137
Riboflavin (mg)	0.7 ± 0.0	0.8 ± 0.0	0.8 ± 0.0	0.045	0.8 ± 0.0	0.8 ± 0.0	0.8 ± 0.0	0.194
Niacin (mg)	6.5 ± 0.1	6.7 ± 0.1	6.7 ± 0.1	0.343	6.7 ± 0.1	6.6 ± 0.1	6.6 ± 0.1	0.597
Vitamin C (mg)	28.8 ± 1.4	28.2 ± 1.5	30.4 ± 1.8	0.550	37.4 ± 1.5	37.0 ± 1.7	37.6 ± 0.19	0.974

^a^Nutrient intake was presented as the amount per 1,000 kcal of energy intake. ^b^The tertile of alternative n-6 to n-3 fatty acid ratio intake. T1: 1st tertile, T2: 2nd tertile, T3: 3rd tertile. ^c^Data is presented as Mean ± SE. ^d^*P*-value is for the test of difference in means of the variables by tertiles of n-6 to n-3 fatty acid ratio using one-way ANOVA (Analysis of Variance) for continuous variables

### Association between dietary fats and AMD

The odds of AMD as a function of the daily omega-6 to omega-3 fatty acid ratio are presented in Table 4. After adjusting for all covariates, women in the highest omega-6 to omega-3 fatty acid ratio tertile exhibited a significantly increased AMD risk compared with those in the lowest (model 2 OR = 1.364, 95% CI = 1.015–1.831). For men, however, there was no significant association between the omega-6 to omega-3 fatty acid ratio and AMD (model 1 OR = 1.141, 95% CI = 0.829–1.570; model 2 OR = 0.995, 95% CI = 0.710–1.395).

**Table 4:** Odds ratios with 95% confidence intervals of tertile of dietary omega-6 to omega-3 fatty acid ratio and AMD

Variables	Men				Women			
	Tertile 1 (*n* = 648)	Tertile 2 (*n* = 648)	Tertile 3 (*n* = 648)	*P*-trend^c^	Tertile 1 (*n* = 864)	Tertile 2 (*n* = 864)	Tertile 3 (*n* = 864)	*P*-trend
Dietary omega-6 to omega-3 fatty acid ratio	2.7 ± 0.0	5.8 ± 0.0	10.9 ± 0.4	< 0.001	2.5 ± 0.0	5.6 ± 0.0	10.1 ± 0.2	< 0.001
Unadjusted	1.00 (Reference)	1.046 (0.717–1.525)	1.138 (0.833–1.556)	0.418	1.00 (Reference)	1.312(1.002–1.719)	1.209(0.916–1.595)	0.177
Model 1^a^	1.00 (Reference)	1.055(0.721–1.545)	1.141(0.829–1.570)	0.418	1.00 (Reference)	1.314(1.000-1.726)	1.232(0.932–1.629)	0.139
Model 2^b^	1.00 (Reference)	0.944(0.644–1.383)	0.995(0.710–1.395)	0.980	1.00 (Reference)	1.359(1.024–1.805)	1.364(1.015–1.831)	0.036

^a^ Model 1: adjusted for age, body mass index, menopausal status (women only). ^b^ Model 2: age, BMI, monthly family income, education level, marital status, disease diagnoses, menopausal status (women only), stress status, alcohol consumption, smoking status, physical activity status, supplement usage, energy intake, beta-carotene intake, and vitamin C intake. ^c^*P*-trend is from multiple regression analysis between n-6 to n-3 fatty acid ratio and multiple adjusted models

## Discussion

In this study, we investigated the relationship between the dietary omega-6 to omega-3 fatty acid ratio and AMD in Koreans aged 50 years or older. Although no association was found between the intake of individual fatty acids, such as omega-6 and omega-3, and AMD, a significant positive association was observed between the omega-6 to omega-3 fatty acid ratio and AMD risk in Korean women.

The fact that high omega-6 to omega-3 fatty acid ratios were significantly associated with higher AMD risk only in women may be due to hormonal differences, particularly the influence of estrogen [48]. Estrogen has protective effects under oxidative stress through its modulation of antioxidant pathways. Post-menopausal women, however, experience a decline in estrogen levels, potentially increasing their vulnerability to oxidative damage and inflammation [48, 49]. In addition, lifestyle and dietary habits may differ between genders, with women in this study reporting higher intakes of omega-6-rich processed foods and lower omega-3-rich seafood consumption.

Oxidative stress plays a critical role in the pathogenesis of AMD [2]. The macula, the center of the retina, is particularly susceptible to oxidative stress due to its exposure to bright light and its high metabolic activity, which is more than twice that of brain nerve tissue [2]. Due to their polyunsaturated structure, PUFAs are highly vulnerable to oxidation, and thus, PUFAs in the retina are particularly vulnerable. As the retina ages, the amount of untreated reactive oxidative stress increases, leading to weakened antioxidant defense mechanisms and increased reactive oxygen species levels [3]. This oxidative stress induces low levels of chronic inflammation in AMD patients. Continuous inflammation leads to the formation of autoantibodies and immune complexes, causing drusen formation and neovascularization in the Bruch’s membrane, as well as macrophage infiltration in the choroid membrane [3]. This process exacerbates neovascularization and ultimately contributes to the progression of AMD.

The association between dietary PUFA intake and AMD risk remains controversial [33–39]. Several cohort studies [24, 33–36] have reported that the dietary intake of omega-3 is associated with a reduced risk of AMD. The Blue Mountains Eye Study found that high omega-3-rich fish consumption reduced the risk of late AMD [33], and the Women’s Health Study observed that DHA and EPA intake were associated with a lower risk of AMD [24]. A 2013 meta-analysis showed that a high dietary intake of omega-3 fatty acids lowered the risk of late AMD by 38% [23]. However, the Age-Related Eye Disease Study 2 (AREDS2), a large multicenter clinical trial supported by the National Institutes of Health, did not demonstrate any preventative effects against AMD through omega-3 supplementation alone [35]. The relationship between omega-6 fatty acids and AMD risk is less well studied [22, 29]. Seddon et al. [22] reported that the intake of nuts rich in omega-6 potentially decreases the risk of AMD progression. In contrast, a study by Cougnard-Grégoire et al. [29] did not find any statistically significant association between the regular consumption of omega-6-rich oils and AMD, regardless of the stage of the disease’s progression.

In our study, we found no association between the individual intakes of each PUFA, omega-3 or omega-6, and AMD (Supplemental Table 1), but a significant positive association between the omega-6 to omega-3 fatty acid ratio and AMD risk in women. An imbalance in the omega-6 and omega-3 levels in the body significantly impacts oxidative stress levels [46]. Previous studies have shown that a high omega-6 to omega-3 fatty acid ratio is associated with increased lipid peroxidation, producing 4-hydroxynonenal and various other oxidative products that contribute to cellular damage in the retina, promoting the progression of AMD [46]. A diet high in omega-6 fatty acids and low in omega-3 fatty acids not only increases oxidative stress but also downregulates protective mechanisms against inflammation and oxidative damage [46]. High omega-6 to omega-3 fatty acid ratios are known to favor the production of pro-inflammatory eicosanoids from arachidonic acid, an omega-6 metabolite, contributing to oxidative stress [45]. In contrast, omega-3 fatty acids are precursors for anti-inflammatory mediators such as resolvins and protectins, which are crucial for resolving inflammation. This imbalance exacerbates retinal oxidative damage, leading to cellular dysfunction in the macula, thereby increasing AMD risk [38]. Furthermore, the retina’s high metabolic activity makes it particularly susceptible to oxidative stress, which could be amplified by a diet skewed toward omega-6 fatty acids [50].

The effects of this disparity in dietary fatty acid compositions underscores the importance of understanding how specific dietary factors impact AMD risk across different populations. A high omega-6 to omega-3 fatty acid ratio is commonly associated with Western cultures’ dietary patterns [42, 44]. For example, the ratios are approximately 17:1 in the United States and 15:1 in the UK and Northern Europe, which contrast sharply with the 4:1 ratio found in regions such as Japan [42]. Traditionally, Asian diets, including those in Korea, are characterized by a higher consumption of fish, a rich source of omega-3 fatty acids. In contrast, Western dietary patterns often exhibit imbalanced omega-6 to omega-3 fatty acid ratios due to an increased consumption of processed foods and oils rich in omega-6 fatty acids [42]. Although there is no universal agreement on the ideal omega-6 to omega-3 fatty acid ratio, a general recommendation is 4:1 [43, 51]. In our study, omega-6 to omega-3 fatty acid ratio intake was classified into tertiles: the average ratio value was 2.6 for the first tertile, 5.7 for the second tertile, and 10.5 for the third tertile. When classified by gender, the third tertile value was consistently about four times that of the first tertile. Traditional Korean diets are rich in omega-3 fatty acids due to a higher consumption of seafood, seaweed, and plant-based foods, as well as fermented foods like kimchi and doenjang. These foods provide antioxidants and anti-inflammatory compounds that may offer protective effects against AMD. Kang and Kim [52] reported the AMD prevalence in Korea and highlighted the role of dietary patterns in disease risk. They found that rates of early AMD were 6.8% and late AMD were 0.6%, significantly lower than those reported in Western countries. These findings illustrate the potential protective effects of traditional Korean diets. Similarly, research by Jang et al. [53] using the Mediterranean Diet Score in Korean elderly populations emphasized the importance of nutrient-dense, anti-inflammatory diets in mitigating AMD risk. However, the gradual Westernization of diets in Korea, evidenced in this study by increasing intakes of omega-6-rich processed foods, could shift this trend, emphasizing the need for public health strategies to preserve traditional dietary practices.

Western-based studies have extensively investigated the association between elevated omega-6 to omega-3 fatty acid ratios and chronic diseases, including AMD, but similar research in Asian populations has been comparatively scarce [47]. This discrepancy likely stems from differing dietary habits between regions, highlighting the need for region-specific studies elucidating these associations. Traditionally, Asian diets, including Korean diets, have been characterized by higher seafood consumption, resulting in lower omega-6 to omega-3 ratios compared to Western diets. However, as dietary habits in Korea continue shifting towards Westernized patterns, the omega-6 to omega-3 ratio has increased, making it an especially relevant factor to consider in relation to chronic diseases such as AMD. In this study, we focused on dietary patterns and their epidemiological associations rather than biomarkers such as the Omega-3 Index [54]. While some have debated the utility of the omega-6 to omega-3 ratio in nutritional research [54], this metric remains widely used in epidemiological studies to reflect the overall dietary balance between these fatty acid types [55]. This ratio serves as a surrogate indicator of broader dietary shifts, particularly the transition towards Westernized eating patterns in Asian populations [4, 21, 46, 55]. Given the scope of this study, the omega-6 to omega-3 ratio is a suitable metric for assessing the dietary context of AMD risk. Notably, some studies in Asian countries have shown a correlation between AMD and the omega-6 to omega-3 fatty acid ratio [4, 21, 46]. However, it has been hypothesized that such results are less frequently found because the rate of fish consumption is higher in Asian countries than in Western countries, a hypothesis previously mentioned in a Japanese study [47]. Although focusing on individual EPA, DHA, or linoleic acid intake could provide additional mechanistic insights, such an approach would not fully capture broader dietary patterns. Our study underscores the nuanced influence of nutrient ratios on AMD risk, even in the absence of direct associations between individual nutrients and AMD. By emphasizing the importance of considering the omega-6 to omega-3 fatty acid ratio alongside individual PUFA intakes, our findings highlight the multifaceted nature of dietary influences on AMD risk.

This study had several limitations. First, as a cross-sectional study, our findings are limited in establishing causality between dietary omega-6 to omega-3 fatty acid ratios and their association with AMD. This study design eliminates the possibility of establishing temporal relationships between dietary intake and disease development. Future longitudinal studies and randomized controlled trials will be necessary to validate these associations and investigate potential mechanisms. Second, omega-6 to omega-3 fatty acid ratios were not measured directly but calculated using the values of omega-6 fatty acid and omega-3 fatty acid in the published data. Third, the dietary assessment in this study used the 24-hour recall method, which, while practical for large-scale research, has inherent limitations. These include potential recall bias and increased variability in the data due to the variability of food intake between days, meaning the reported quantities may not accurately reflect habitual dietary patterns. Future studies employing food frequency questionnaires (FFQs) or multi-day dietary records would provide more reliable estimates of regular dietary intakes and offer a clearer picture of omega-6 and omega-3 fatty acid consumption patterns. Fourth, data limitations prevented us from analyzing AMD risk based on stage (early, intermediate, or advanced). Fifth, a lack of detailed information on omega-3 supplement types and dosages, as well as the absence of genetic data, limited our ability to perform more sensitive analyses. Future studies incorporating comprehensive supplement data and genetic information will help provide a more precise understanding of these associations. Despite these limitations, as a nationwide sample of middle-aged Koreans, the study benefits from its representativeness, providing generalizable insights into the association between dietary fatty acid ratios and AMD within this population.

## Conclusions

This study highlights a potential association between high omega-6 to omega-3 fatty acid ratios and increased AMD risk among Korean women aged 50 and older. This finding suggests that considering nutrient ratios, rather than individual nutrients alone, may offer valuable insights for AMD prevention and public health policy development. Targeted dietary interventions aimed at achieving balanced omega-6 to omega-3 ratios, particularly for post-menopausal women, could serve as an effective strategy for addressing this growing health concern. Further research with larger sample sizes and prospective or interventional designs is warranted to deepen our understanding of this relationship and inform future preventive strategies.

## Electronic supplementary material

Below is the link to the electronic supplementary material.

Supplementary Material 1

## Data Availability

The datasets, code book, and analytic code analyzed in the current study are available from the corresponding author upon reasonable request.

## Abbreviations

AMD: Age-related macular degeneration
DHA: Docosahexaenoic acid
EPA: Eicosapentaenoic acid
KNHANES: Korea national health and nutrition examination survey
SFA: Saturated fatty acid
MUFA: Monounsaturated fatty acid
PUFA: Polyunsaturated fatty acid
BMI: Body mass index
