# Correction: Association between the dietary omega-6 to omega-3 fatty acid ratio and age-related macular degeneration in Korean adults

**DOI:** 10.1186/s12937-025-01140-6

**Published:** 2025-04-29

**Authors:** Won Jang, Yuna Kim, Hyesook Kim

**Affiliations:** 1 Department of Food and Nutrition, Wonkwang University, 460 Iksandae-ro, Iksan, Jeonbuk-do Republic of Korea; 2 Institute for Better Living, Wonkwang University, 460 Iksandae-ro, Iksan, Jeonbuk-do Republic of Korea; 3 Department of Clinical Nutrition Science, The Graduate School of Clinical Health Sciences, Ewha Womans University, 52 Ewhayeodae-gil, Seodaemun-gu, Seoul, Republic of Korea

**Correction: Nutr J 24**,** 29 (2025)**

10.1186/s12937-025-01090-z

Following publication of the original article [1], the author reported errors as follows:

1. In **Table 1**,** 2**,** and 3**, the term **“SD”** in the legend should be corrected to **“SE”**.
2. In **Table 3**, the legend **“Nutrients using logistic regression analysis after calculating energy intake per 1000 kcal.”** should be revised to **“Nutrient intake was presented as the amount per 1**,**000 kcal of energy intake.”**.
3. In **Table 3**, the duplicated **“EPA(mg)”** should be removed.
4. In **Table 3**, the **“Omega 6: Omega 3 Ratio”** may be misinterpreted as a simple ratio rather than an energy-adjusted ratio. To avoid confusion, This should be removed.

The original article has been updated.
