# Supplementary material for: Association between the dietary omega-6 to omega-3 fatty acid ratio and age-related macular degeneration in Korean adults
Source: Nutr J. 2025 Mar 9;24:29. doi: 10.1186/s12937-025-01090-z (PMC11892259; doi:10.1186/s12937-025-01090-z)
Supplement: Supplementary file 1 — Supplementary Material 1 [file 12937_2025_1090_MOESM1_ESM.docx]

**Association between the dietary omega-6 to omega-3 fatty acid ratio and age-related macular degeneration in Korean adults**

Won Jang^1,2^, Yuna Kim^3^, Hyesook Kim^1,2*^

^1^Department of Food and Nutrition, Wonkwang University, Iksan, Korea

^2^Institute for Better Living, Wonkwang University, Iksan, Korea

^3^Department of Clinical Nutrition Science, The Graduate School of Clinical Health Sciences, Ewha Womans University, Seoul, Republic of Korea

***Correspondence**: Hyesook Kim: Tel.: 82-63-850-6896; hskim81@wku.ac.kr

**Supplemental Table 1.** Odds ratios with 95% confidence intervals of tertile of dietary fatty acids and AMD

| **Variables** | **Men** | | | | **Women** | | | |
| --- | --- | --- | --- | --- | --- | --- | --- | --- |
|  | **Tertile 1 (*n* = 648)** | **Tertile 2 (*n* = 648)** | **Tertile 3 (*n* = 648)** | ***P*-trend^c^** | **Tertile 1 (*n* = 864)** | **Tertile 2 (*n* = 864)** | **Tertile 3 (*n* = 864)** | ***P*-trend** |
| SFA |  |  |  |  |  |  |  |  |
| Median intake, g | 4.4 ± 0.1 | 9.8 ± 0.1 | 21.8 ± 0.4 |  | 3.0 ± 0.1 | 7.5 ± 0.1 | 16.8 ± 0.3 |  |
| Model 1^a^ | 1.00 (Ref) | 0.92 (0.67–1.26) | 0.97 (0.69–1.36) | 0.733 | 1.00 (Ref) | 0.86 (0.65–1.15) | 1.10 (0.83–1.47) | 0.105 |
| Model 2^b^ | 1.00 (Ref) | 1.00 (0.71–1.43) | 0.84 (0.60–1.18) | 0.369 | 1.00 (Ref) | 0.87 (0.64–1.18) | 1.07 (0.79–1.45) | 0.258 |
| MUFA |  |  |  |  |  |  |  |  |
| Median intake, g | 3.9 ± 0.1 | 9.6 ± 0.1 | 22.8 ± 0.5 |  | 2.7 ± 0.1 | 7.3 ± 0.1 | 17.0 ± 0.2 |  |
| Model 1 | 1.00 (Ref) | 0.99 (0.73–1.34) | 1.07 (0.79–1.44) | 0.661 | 1.00 (Ref) | 0.88 (0.64–1.19) | 1.14 (0.85–1.53) | 0.124 |
| Model 2 | 1.00 (Ref) | 1.04 (0.73–1.47) | 0.89 (0.64–1.23) | 0.974 | 1.00 (Ref) | 0.94 (0.67–1.30) | 1.16 (0.84–1.60) | 0.277 |
| PUFA |  |  |  |  |  |  |  |  |
| Median intake, g | 4.3 ± 0.1 | 9.1 ± 0.1 | 19.3 ± 0.3 |  | 3.0 ± 0.0 | 7.1 ± 0.1 | 15.6 ± 0.3 |  |
| Model 1 | 1.00 (Ref) | 0.89 (0.65–1.23) | 0.99 (0.71–1.37) | 0.521 | 1.00 (Ref) | 0.78 (0.58–1.05) | 1.01 (0.74–1.39) | 0.157 |
| Model 2 | 1.00 (Ref) | 0.99 (0.69–1.41) | 0.97 (0.68–1.38) | 0.974 | 1.00 (Ref) | 0.83 (0.60–1.15) | 1.05 (0.74–1.49) | 0.277 |
| Omega-3 FA |  |  |  |  |  |  |  |  |
| Median intake, g | 0.6 ± 0.0 | 1.4 ± 0.0 | 3.9 ± 0.1 |  | 0.4 ± 0.0 | 1.1 ± 0.0 | 3.4 ± 0.1 |  |
| Model 1 | 1.00 (Ref) | 1.10 (0.81–1.50) | 1.02 (0.73–1.42) | 0.537 | 1.00 (Ref) | 1.05 (0.77–1.42) | 1.09 (0.81–1.46) | 0.845 |
| Model 2 | 1.00 (Ref) | 1.10 (0.78–1.54) | 1.08 (0.75–1.56) | 0.857 | 1.00 (Ref) | 1.02 (0.74–1.41) | 1.00 (0.71–1.39) | 0.987 |
| Omega-6 FA |  |  |  |  |  |  |  |  |
| Median intake, g | 4.9 ± 0.2 | 8.7 ± 0.2 | 13.9 ± 0.3 |  | 3.4 ± 0.1 | 6.9 ± 0.1 | 10.8 ± 0.3 |  |
| Model 1 | 1.00 (Ref) | 1.06 (0.76–1.47) | 1.08 (0.79–1.46) | 0.890 | 1.00 (Ref) | 1.51 (1.14–1.99) | 1.33 (0.97–1.82) | 0.014 |
| Model 2 | 1.00 (Ref) | 0.94 (0.64–1.37) | 0.96 (0.64–1.44) | 0.945 | 1.00 (Ref) | 1.48 (1.8–2.02) | 1.31 (0.89–1.94) | 0.050 |
| ^a^ Model 1: adjusted for age, BMI , menopausal status(women only)  ^b^ Model 2: age, BMI, monthly family income, education level, marital status, disease diagnoses, menopausal status (women only), stress status, alcohol consumption, smocking status, physical activity status, supplement usage, energy intake, beta-carotene intake, and vitamin C intake | | | | | | | | |

^c^ *P*-trend is from multiple regression analysis
